# Design of polar self-assembling lactic acid derivatives possessing submicrometre helical pitch

**DOI:** 10.3762/bjnano.9.33

**Published:** 2018-01-29

**Authors:** Alexej Bubnov, Cyril Vacek, Michał Czerwiński, Terezia Vojtylová, Wiktor Piecek, Věra Hamplová

**Affiliations:** 1 Institute of Physics, The Czech Academy of Sciences, Prague, 18221, Czech Republic; 2 Gymnázium U Balvanu, Jablonec nad Nisou, 46634, Czech Republic; 3 Faculty of Advanced Technologies and Chemistry, Military University of Technology, Warsaw, Poland

**Keywords:** ferroelectric liquid crystal, keto group, self-assembly on the nanoscale, soft ferroelectrics, submicrometre helical pitch length

## Abstract

Several new lactic acid derivatives containing the keto linkage group far from the chiral part and short alkyl chains have been synthesized and characterised by polarising optical microscopy, differential scanning calorimetry, as well as electro-optic and dielectric spectroscopy. The materials possess a self-assembling behaviour on the nanoscale level as they form polar smectic liquid crystalline mesophases, namely the orthogonal paraelectric SmA* and the tilted ferroelectric SmC* phases, in a broad temperature range down to room temperature. A short helical pitch (≈120–320 nm), relatively high spontaneous polarisation (≈150 nC/cm^2^) and reasonable tilt angle values have been determined within the temperature range of the tilted ferroelectric SmC* phase. The obtained results make the new materials useful for the advanced mixture design and for further utilisation in electro-optic devices based on the deformed helix ferroelectric effect.

## Introduction

The design of advanced self-assembling materials possessing specific properties still remains a highlighted current challenge for the scientific community due to the huge potential for utilisation of such functional materials for various applications in electro-optics and photonics [1–5]. Chiral materials possessing the polar smectic liquid crystalline (LC) mesophases (e.g., layered structure on nanoscale) belongs to one of the most exciting but special groups of such smart self-organized organic materials [1–2,6]. These advanced materials are able to self-assemble at nanoscopic and mesoscopic length scales; the intermolecular interactions that take place and are responsible for the self-organisation behaviour can be accurately adjusted by a specific molecular design, for example, by creating the mesogenic molecule using various structural or functional blocks [7–8] or even further tuned by the design of advanced LC multicomponent mixtures or LC nanocomposites. Chiral self-assembling materials possessing smectic phases with ferroelectric properties on the nanoscale, specifically the lactic acid derivatives [9–15], attract considerable attention [16–19]. There are several considerable advantages of the lactic acid derivatives as a subclass of chiral self-assembling materials that makes them attractive [20], namely (i) the occurrence of a broad variety of basic LC phases, including the cholesteric, paraelectric, ferroelectric and antiferroelectric smectic phases as well as frustrated phases like the twist grain boundary – TGBA* and TGBC* phases or cubic SmQ* phase and re-entrant orthogonal and tilted phases; (ii) the utilisation of the lactic unit as a precursor of chiral centre minimises the synthetic cost with respect to the most commonly used chiral precursors; (iii) the melting points in the range of 5–70 °C are often desirable for application needs and the LC phases can easily be supercooled well below room temperature; and (iv) the lactic acid derivatives usually show no aging and are highly thermally as well as chemically stable. Due to the properties mentioned above, lactic acid derivatives demonstrate their high ability to be used as smart and functional dopants for the design and tailoring of the advanced multicomponent LC mixtures [21–25] and LC composite materials [25–32] and tuning their properties.

It is quite obvious that the weakening of the longitudinal dipole moment of the molecule results in relative enhancement of the transversal dipole moment close to the chiral centre that can give rise to a considerable stabilisation of smectic phases [33–34]. Specifically, the ester and keto linkage groups placed on the opposite sides of the biphenyl unit within the molecular core can almost compensate for the longitudinal dipole moment of this part of the mesogenic molecule, while only a minor contribution caused by the polar groups on the last phenyl ring remains almost uncompensated [9]. The replacement of the ether or ester groups (or their combination) by a keto group in order to link alkyl chains with the molecular core can result in a considerable increase in the values of the spontaneous polarisation [35–36]. Specifically, several series of the lactic acid derivatives [9] possessing a keto group utilised as a linkage between the non-chiral terminal alkyl chain, the molecular core [9,20,37–39] and the chiral part based on one [20,37–38], two [40] or three [9,39] lactate units have been intensively studied during last years.

It has been observed that for these specific materials with one chiral lactate unit a decrease of the alkyl chain lengths at the chiral carbon and attached at the non-chiral terminal part can result in substantial decrease of the helical pitch length [20,37–38].

Recently, a deformed helix ferroelectric (DHF) effect [5,41–44] discovered in smectic liquid crystalline materials became a focus of interest of the scientific community due to its application potential. Generally, this effect is characterised by a tunable, continuous, and hysteresis-free optical phase shift that operates at low voltages. By using the DHF effect, the possibility for realising specific displays, effective light shutters, voltage sensors (with exceptionally fast response and near-perfect linearity and low-cost), and tuneable telecommunication optical fibres [45–46] is very encouraging [5,44]. However, requirements for materials that perform at the DHF mode are usually very complex, which multicomponent mixtures [44,47–50] only can fulfil.

The main objective of this work is to design new materials and to look for the structure–property relationship of specific lactic acid derivatives containing one lactate group and the keto linkage group attached to the molecular core far from the chiral centre. A crucial issue addressed in the present work is to achieve materials possessing a broad temperature range of the ferroelectric SmC* phase down to room temperatures and sub-micrometre (order of hundred nanometres) values of the helical pitch. Such materials can be potentially utilised for the design of advanced mixtures for the electro-optic and photonic devices, specifically those based on the DHF effect. The material properties will be discussed also in terms of molecular architecture–mesomorphic property relationship.

## Results and Discussion

### Design and synthesis

**Scheme 1:**
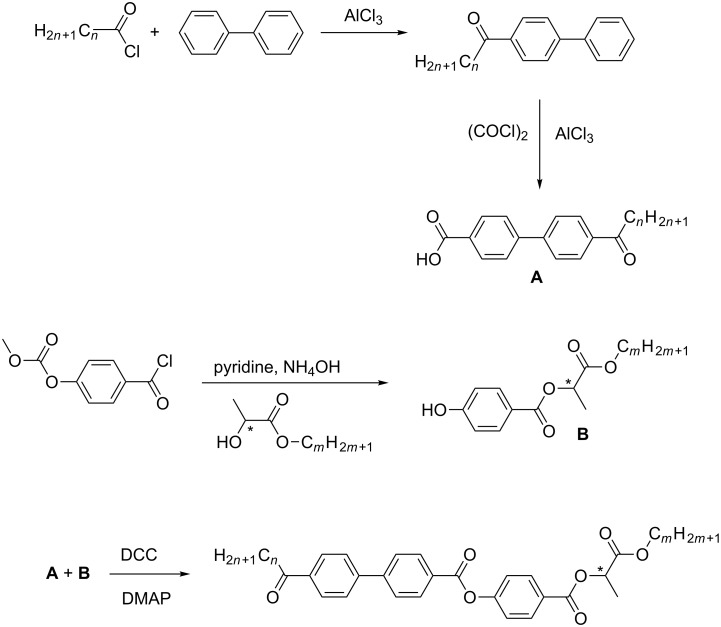
Reaction scheme for the synthesis of new (S)-4-((1-alkoxy-1-oxopropan-2-yloxy)carbonyl)phenyl 4'-alkyloylbiphenyl-4-carboxylates denoted as KL *n*/*m* series.

The syntheses of new self-assembling materials (denoted as KL *n*/*m*, where *n* is the number of carbon atoms in the non-chiral alkyl chain and *m* is the number of the carbon atoms in the chiral short alkyl unbranched chain, namely the (*S*)-4-((1-alkoxy-1-oxopropan-2-yloxy)carbonyl)phenyl 4'-alkyloylbiphenyl-4-carboxylates) were performed similarly as generally described in [37]. The specific reaction scheme for the synthetic design is presented in Scheme 1.

The crude products were purified by column chromatography on silica gel (Kieselgel 60, Merck, Darmstadt) using a mixture of CH_2_Cl_2_ and acetone (99.8:0.2) as the eluent and recrystallized from methanol. The molecular structure was checked for all synthesised compounds by ^1^H NMR spectra recorded on a Varian VNMRS 300 instrument, where deuteriochloroform (CDCl_3_) served as a solvent. The signal of the solvent was used as an internal standard. The purity of the final compounds was checked by HPLC analysis (high-pressure pump ECOM Alpha; column WATREX Biospher Si 100, 250 × 4 mm, 5 µm; detector WATREX UVD 250) and were found to be >99.8% with the mixture of 99.9% of toluene and 0.1% of methanol as an eluent using a UV–vis detector (λ = 290 nm). The optical rotation 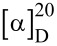 was measured in chloroform solution (*c* = 0.10) using a polarimeter from Optical Activity Ltd., Ramsey, UK. The optical rotation was found as follows for **KL 3/4**: 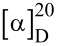 +16.0; **KL 3/5**: 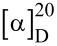 +15.0; **KL 4/4**: 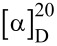 +18.9; **KL 4/5**: 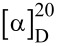 +13.5; **KL 4/6**: 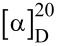 +14.1.

^1^H NMR for **KL 3/4** (CDCl_3_, 300 MHz) 8.29 (d, 2H, ortho to -COOAr), 8.18 (d, 2H, ortho to -COOC*), 8.07 (d, 2H, ortho to -CO-), 7.76 (m, 4H, ortho to -Ar), 7.34 (d, 2H, ortho to -OCO), 5.34 (q, 1H, C*H), 4.16 (m, 2H, COOCH_2_), 3.00 (t, 2H, CH_2_CO), 1.64 (d, 3H, CH_3_C*), 1.3–1.7 (m, 6H, CH_2_), 0.87 (m, 6H, CH_3_).

^1^H NMR for **KL 3/5** (CDCl_3_, 300 MHz) 8.29 (d, 2H, ortho to -COOAr), 8.19 (d, 2H, ortho to -COOC*), 8.07 (d, 2H, ortho to -CO-), 7.76 (m, 4H, ortho to -Ar), 7.34 (d, 2H, ortho to -OCO), 5.36 (q, 1H, C*H), 4.16 (m, 2H, COOCH_2_), 3.00 (t, 2H, CH_2_CO), 1.62 (d, 3H, CH_3_C*), 1.3–1.7 (m, 8H, CH_2_), 0.87 (m, 6H, CH_3_).

^1^H NMR for **KL 4/4** (CDCl_3_, 300 MHz) 8.29 (d, 2H, ortho to -COOAr), 8.18 (d, 2H, ortho to -COOC*), 8.06 (d, 2H, ortho to -CO-), 7.75 (m, 4H, ortho to -Ar), 7.34 (d, 2H, ortho to -OCO), 5.35 (q, 1H, C*H), 4.14 (m, 2H, COOCH_2_), 3.00 (t, 2H, CH_2_CO), 1.63 (m, 3H, CH_3_C*), 1.3–1.7 (m, 8H, CH_2_), 0.88 (m, 6H, CH_3_).

^1^H NMR for **KL 4/5** (CDCl_3_, 300 MHz) 8.29 (d, 2H, ortho to -COOAr), 8.19 (d, 2H, ortho to -COOC*), 8.07 (d, 2H, ortho to -CO-), 7.77 (m, 4H, ortho to -Ar), 7.34 (d, 2H, ortho to -OCO), 5.34 (q, 1H, C*H), 4.15 (m, 2H, COOCH_2_), 3.00 (t, 2H, CH_2_CO), 1.63 (d, 3H, CH_3_C*), 1.3–1.7 (m, 10H, CH_2_), 0.88 (m, 6H, CH_3_).

^1^H NMR for **KL 4/6** (CDCl_3_, 300 MHz) 8.30 (d, 2H, ortho to -COOAr), 8.19 (d, 2H, ortho to -COOC*), 8.06 (d, 2H, ortho to -CO-), 7.75 (m, 4H, ortho to -Ar), 7.33 (d, 2H, ortho to -OCO), 5.35 (q, 1H, C*H), 4.14 (m, 2H, COOCH_2_), 3.00 (t, 2H, CH_2_CO), 1.63 (d, 3H, CH_3_C*), 1.3–1.7 (m, 12H, CH_2_), 0.87 (m, 6H, CH_3_).

### Mesomorphic behaviour

**Figure 1:**
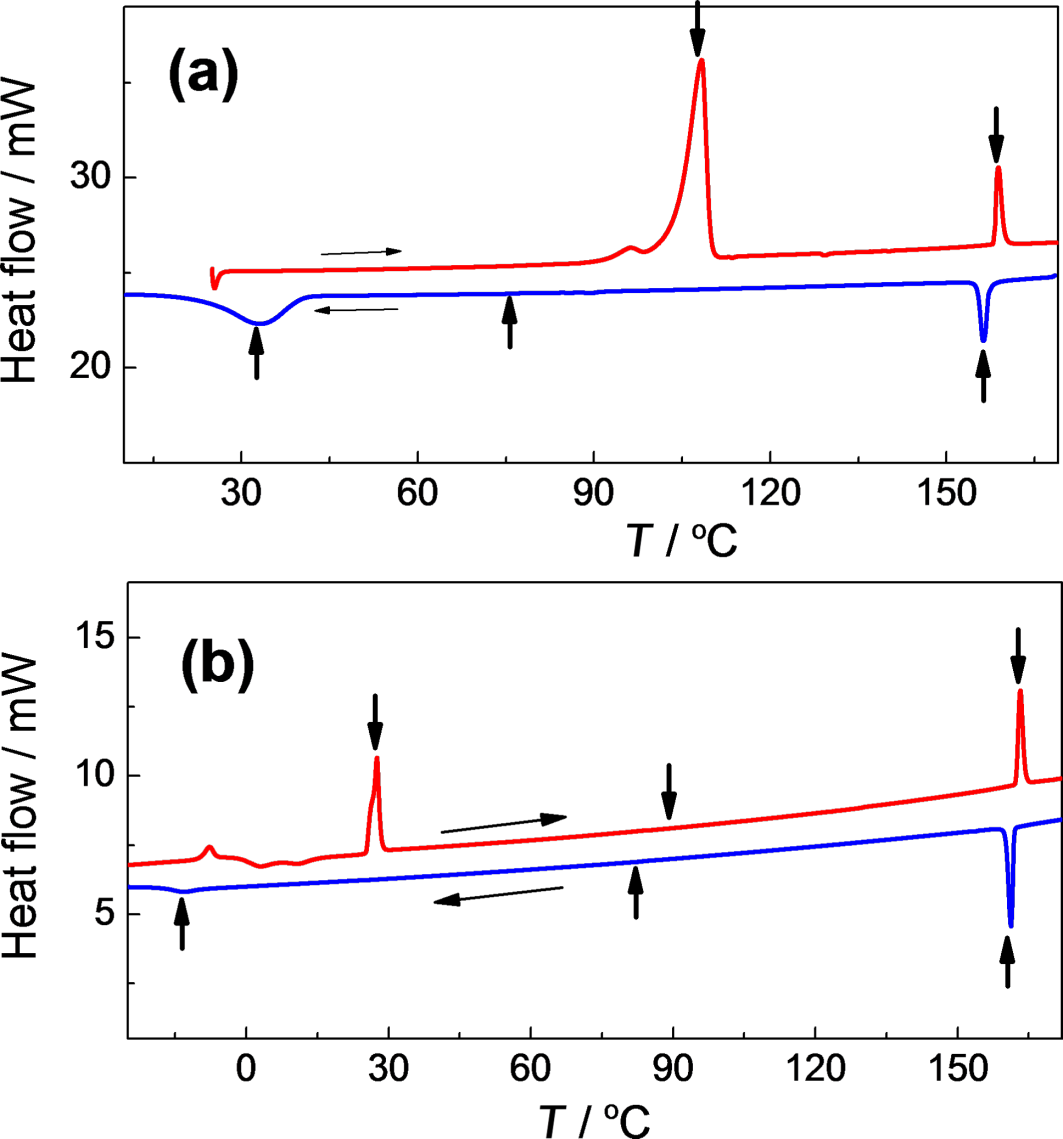
Differential scanning calorimetry (DSC) plot of heating/cooling runs (indicated by horizontal arrows) for **KL3/4** (a) and **KL 4/5** (b). Vertical arrows indicate the peaks corresponding to phase transitions.

The mesomorphic properties of the studied compounds KL *n*/*m* are summarised in Table 1. The differential scanning calorimetry (DSC) heating/cooling runs of two selected compounds, as examples, are presented in Figure 1. All compounds exhibit broad temperature ranges of the orthogonal paraelectric SmA* (up to 100 K) and tilted ferroelectric SmC* (up to 90 K) phases observed on cooling from the isotropic phase (Iso). The polar phases are observed down to room temperatures before the onset of the crystal (Cr) phase. The temperature of the Iso–SmA* phase transition slightly increases with the length of the chain attached to the non-chiral molecular terminal element. An increase of the length of such a chain results in broadening of the ferroelectric SmC* phase also, similar to [40]. The melting point as well as the upper temperature of existence of the SmC* phase decreased with increasing length of the alkyl chain at the chiral carbon atom and the same length of non-chiral terminal chain.

**Table 1:** Sequence of phases, phase transition temperatures *T* [°C], transition enthalpies Δ*H* [J/g] (measured on cooling with DSC [5 K min^−1^]) and melting points mp [°C] (measured on heating) for all studied compounds. (“•” indicates that the phase exists, value in brackets indicates that the phase transition has not been determined by differential scanning calorimetry but by polarising optical microscopy only).

	mp / Δ*H*	Cr	*T* / Δ*H*	SmC*	*T* / Δ*H*	SmA*	*T* / Δ*H*	Iso

**KL 3/4**	108 [+44.0]	•	33 [−23.2]	•	75 [−0.1]	•	156 [−5.9]	•
**KL 3/5**	88 [+42.0]	•	34 [−33.0]	•	51 [−0.01]	•	152 [−5.8]	•
**KL 4/4**	82 [+46.7]	•	6 [−2.6]	•	98 [−0.01]	•	168 [−7.1]	•
**KL 4/5**	28 [+11.2]	•	−13 [−2.5]	•	86 [−0.01]	•	161 [−7.5]	•
**KL 4/6**	−11 [+1.1]	•	−16 [*]	•	75 [−0.01]	•	162 [−7.8]	•

For all compounds the SmC* phase remains quite stable also at its supercooled state, i.e., below the melting point; the crystallisation does not occur even under an applied electric field. Moreover, the uniform alignment of the SmC* phase texture is easily reachable by applying of a low frequency a.c. electric field.

### Spontaneous quantities

**Figure 2:**
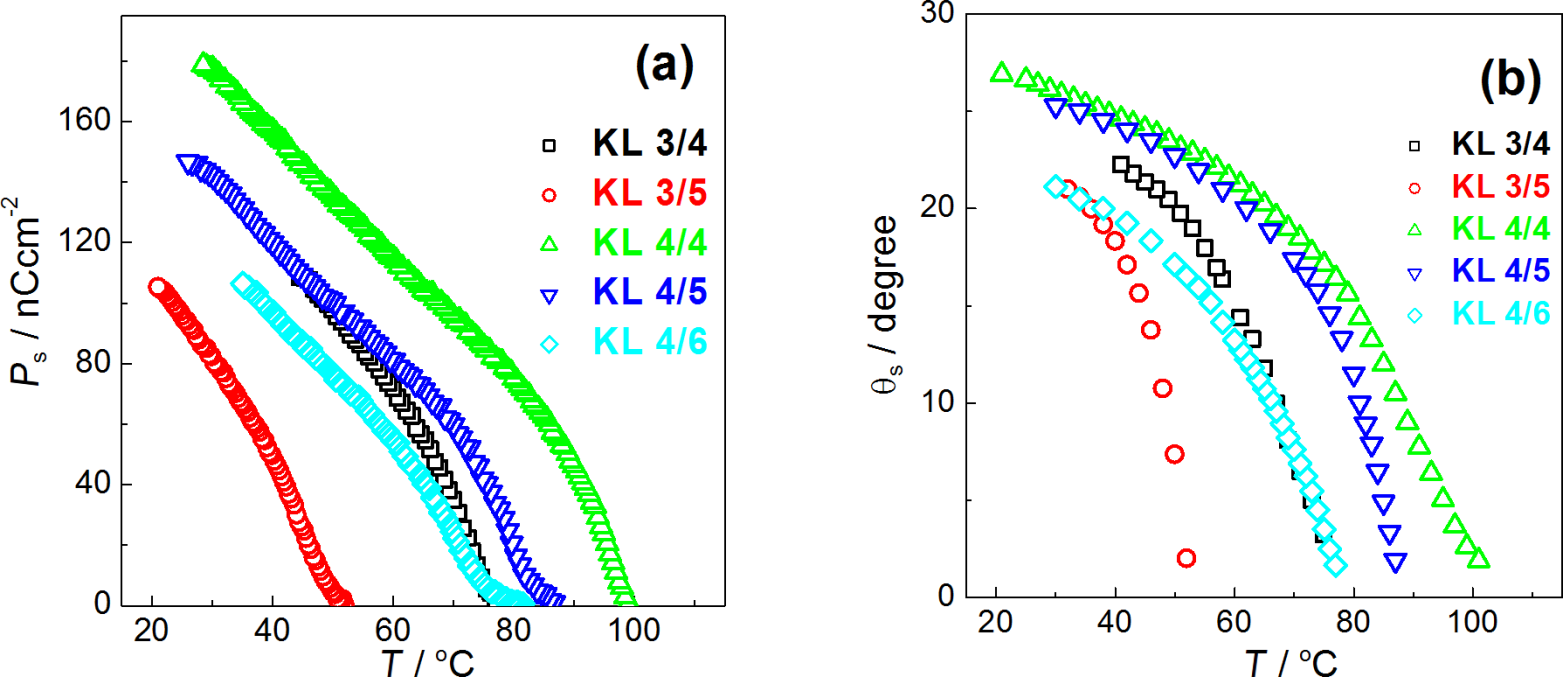
Temperature dependencies of spontaneous polarization *P*_s_, (a) and tilt angle of molecules θ_s_, measured optically (b) for **KL 3/4** (black rectangle), **KL 3/5** (red circle), **KL 4/4** (green triangle), **KL 4/5** (blue triangle) and **KL 4/6** (cyan diamond) as indicated.

The temperature dependences of the spontaneous polarization *P*_s_ and of the tilt angle θ_s_ (angle of (in-layer) molecular director with respect to the smectic layer normal) are shown in Figure 2a,b, respectively. The values of the spontaneous polarization *P*_s_ do not reveal any tendency to saturate on cooling. While comparing the homologues with the same length of non-chiral terminal chain, the *P*_s_ as well as θ_s_ values were found to slightly decrease with the increasing length of the alkyl chain at the chiral carbon atom. On the other side, the increase of the length of the non-chiral terminal chain causes an increase of the *P*_s_ and θ_s_ values for compounds with the same chiral terminal chain (see **KL 3/4** and **KL 4/4** as well as **KL 3/5** and **KL 4/5** in Figure 2a,b). The *P*_s_ reaches the highest value of about 170 nC·cm^−2^ for **KL 4/4** (see Figure 2a). It is a well-known fact that the measured θ_s_ values involve at least two components, namely the real spontaneous tilt angle existing without applied electric field and the field-induced tilt angle existing due to the electro-clinic effect [51]. However, the field-induced component of the tilt angle should be taken into account only in the vicinity of the SmA*–SmC* phase transition. At the SmA*–SmC* phase transition, the increase of *P*_s_ and θ_s_ values of KL *n*/*m* compounds is continuous (see Figure 2ab), which can be related to the second order phase transition. Similar materials with much longer alkyl chains possess this type of spontaneous polarisation and tilt angle behaviour as well [20]. Saturated θ_s_ values reach 20–28°, which is very comparable to values that can be found for materials with the ether linkage group [9,52] or for similar compounds with the keto group attached to longer alkyl chains [20,37].

The electro-optic parameters of the studied compounds are temperature dependent, namely, the spontaneous polarisation and tilt angle, which are extremely important for practical applications [5,44]. However, these parameters can be stabilised by formulation of the multicomponent mixtures which goes beyond the present work.

### Helical pitch length

**Figure 3:**
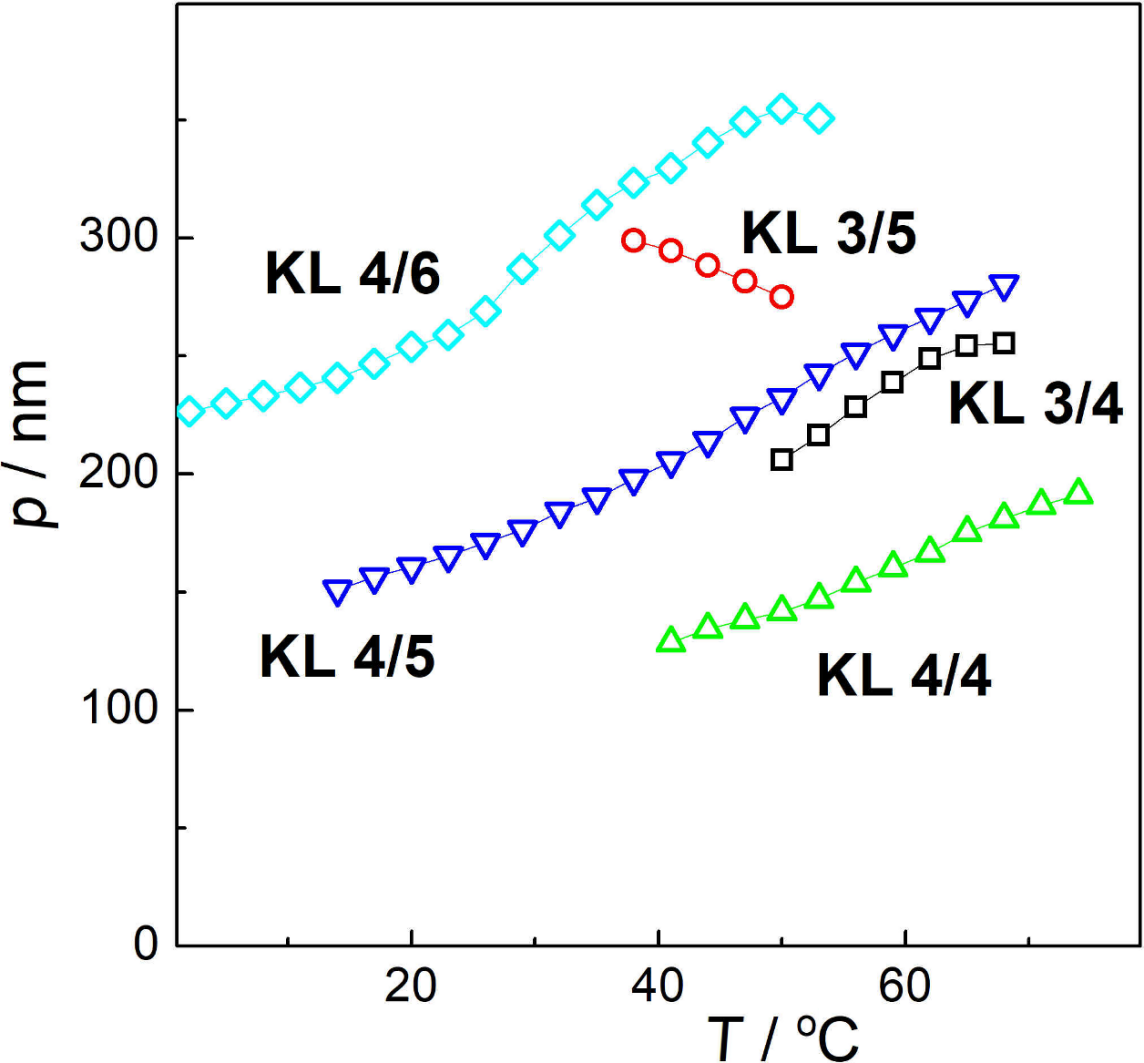
Temperature dependencies of the helix pitch length *p* for **KL 3/4** (black rectangle), **KL 3/5** (red cicle), **KL 4/4** (green triangle), **KL 4/5** (blue triangle) and **KL 4/6** (cyan diamond) as indicated.

The parameters of the helical structure, namely the helix pitch length *p*, were determined on homeotropically aligned samples by measuring the selective light reflection within the temperature range of the tilted ferroelectric SmC* phase. The temperature dependencies of *p* values are presented in Figure 3. All the KL *n*/*m* compounds possess a helical pitch within 120–350 nm, which is a quite low value. The values of the helical pitch slightly decrease with the temperature for all compounds with exception of **KL 3/5** for which a slight increase was observed with the temperature decrease. The difference in the temperature dependence of the helical pitch (**KL 3/5** with respect to other studied compounds) can be associated with a different helical twist sense, which in turn depends on the change of the concentration of different conformers promoting opposite handedness, as described in [49]. The values of the helical pitch length considerably decrease with the decrease of both alkyl chain lengths while comparing to the results obtained on similar compounds [20,37,40].

### Dielectric spectroscopy

**Figure 4:**
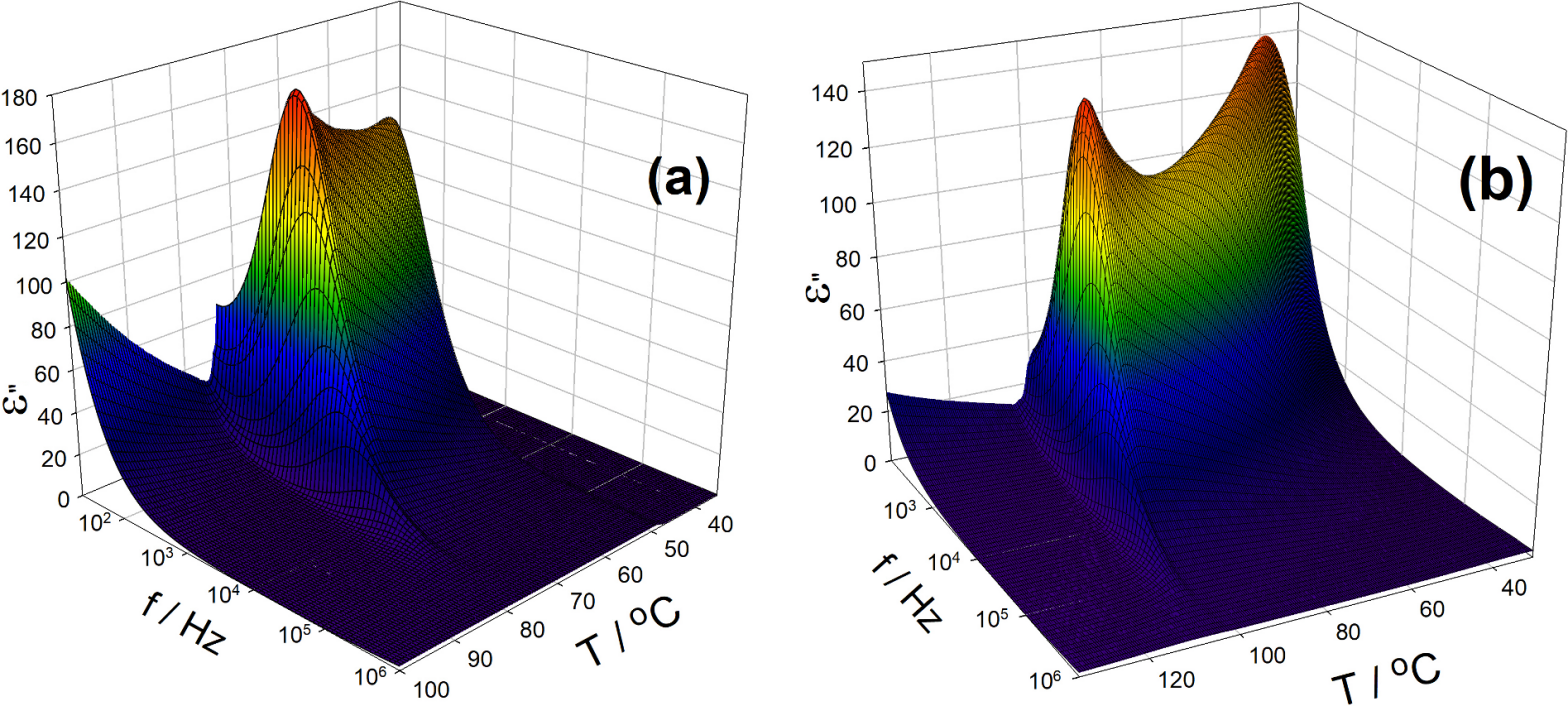
3D plots of the imaginary part of complex permittivity for **KL 3/4** (a) and **KL 4/4** (b) showing strong contributions of the Goldstone mode within the temperature range of the tilted ferroelectric SmC* phase.

For the two selected materials, illustrative results of the imaginary part, ε”, of complex permittivity versus temperature and versus frequency are presented in Figure 4a,b. The dielectric absorption ε” spectra obtained within the whole temperature range of the ferroelectric SmC* phase at zero bias electric field reveal a strong contribution of the Goldstone mode (this relaxation mode is related to azimuthal fluctuations of the molecules in the smectic layer) with a typical relaxation frequency of several kHz. This behaviour fully confirms the ferroelectric character of the SmC* phase detected for all studied materials from the KL *n*/*m* series. The relaxation frequency of the mode slightly decreases with the temperature decrease while the dielectric strength is decreased to a local minimum value and then begins to increase until crystallisation occurs. This is quite typical behaviour for the Goldstone mode at the ferroelectric SmC* phase [39,53–54]. The soft mode behaviour, which is related to fluctuations of molecules (arranged in smectic layers) in the direction of the tilt angle θ_s_, has been detected in the SmA* phase close to the SmA*–SmC* phase transition. A comprehensive discussion on the specific behaviour of all the detected modes, revealed by the broad-band dielectric spectroscopy, with respect to the mixture composition, is beyond the scope of the present work and will be presented elsewhere.

## Conclusion

Several chiral liquid crystalline materials possessing two ester groups in the molecular core and the non-chiral alkyl chain linked by a keto group where shown to exhibit a broad temperature range of the paraelectric orthogonal SmA* and the ferroelectric tilted SmC* phases down to room temperature. These materials contain relatively short chiral and non-chiral alkyl chains with respect to similar compounds designed previously [20,37–38,40,55]. The increase of the non-chiral chain length (*n*) causes a considerable broadening of the temperature range of the ferroelectric SmC* phase. However, the increase of the chiral chain length (*m*) results in a minor broadening of the paraelectric SmA* phase and shift of the ferroelectric SmC* phase down to lower temperatures. The studied compounds possess a relatively high spontaneous polarisation, which reaches values up to 170 nCcm^−2^ without saturation. The values of the spontaneous polarisation and spontaneous tilt angle are slightly dependent on the length of the chiral (*m*) and non-chiral (*n*) chain lengths. However, this effect is not so pronounced with respect to that found for homologues with longer alkyl chains [9,20,37]. The helix pitch length was found within the 120–320 nm range and remains nearly temperature independent. The low values of the helical pitch length obtained for the pure liquid crystalline materials can be considered as the most exciting result reached in this work. The helix pitch slightly increased with the number (*n*) of carbon atoms in the non-chiral chain and much more substantially with the number (*m*) of carbon atoms in the chiral chain. The results of the broadband dielectric spectroscopy measurements confirm the ferroelectric character of the polar smectic phase. The soft mode and the Goldstone mode were found within the temperature ranges of the paraelectric SmA* and the ferroelectric SmC* phases, respectively.

The studied compounds with the keto group in the molecular core can be very useful as chiral dopants for advanced mixtures [9,47,56] aimed for specific applications due to the wide temperature range of the ferroelectric SmC* phase down to room temperature and the relatively high spontaneous polarisation. By simply applying an electric field, it is possible to reach uniform alignment which remains very stable with time. Their most exciting property, namely the helical pitch length of 120–320 nm, makes the materials from the KL *n*/*m* series potentially applicable for the design of advanced functional multicomponent mixtures (like in [44,47–50,57–58]) aimed for electro-optic and photonic devices based on the deformed helix ferroelectric effect [5,41–44,59]. Further studies of similar materials are in progress and will be presented elsewhere.

## Experimental

### Sample preparation

The LC materials were filled into glass cells (Military University of Technology, cell gap of 12 μm, homogeneous alignment) with indium tin oxide transparent electrodes and polyimide layers rubbed unidirectional, which ensured a planar geometry supporting an induction of bookshelf-like smectic structures. The alignment was improved by electric field action in the frequency range of 10–20 Hz and an amplitude of 40 kV·cm^−1^, applied for about 5–20 min while cooling from orthogonal to tilted smectic phase. The samples, hermetically sealed in aluminium pans of 3–5 mg, were used for DSC studies.

### Mesomorphic properties

For all studied materials the phase transition temperature and phase sequence were determined by observations of characteristic textures and their changes in the cooling cycle using a polarising optical microscope. The LINKAM LTS E350 heating stage with a TMS-93 temperature programmer was used for the temperature control, which enabled temperature stabilisation within ±0.1 K. The phase transition temperatures were checked by DSC (Perkin-Elmer DSC 8000) on cooling/heating runs at a rate of 5 K·min^−1^ in a nitrogen atmosphere. The temperature and enthalpy change values were calibrated on the extrapolated onset temperatures and the enthalpy changes of the melting points of water, indium and zinc.

### Spontaneous polarisation

The values of the spontaneous polarisation *P*_s_ were determined from the polarisation current peak by driving the sample with a triangular electric field at a frequency of 30 Hz and an electric field magnitude of 10 V/μm. The driving voltage was supplied by an Agilent 33210A function generator amplified with a linear amplifier, providing a maximum amplitude of about ±100 V. The Tektronix DPO4034 digital oscilloscope provided information about the switching current profile versus time.

### Spontaneous tilt angle

The spontaneous tilt angle θ_s_ values have been determined optically using well-aligned samples at bookshelf-like surface-stabilised structures, observing the difference between extinction positions at crossed polarizers under opposite d.c. electric fields ±40 kV·cm^−1^.

### Helix pitch length

The helix pitch length *p* was determined on homeotropically aligned samples under study placed on a single glass plate while leaving the other surface of the sample free. The measurements of the helical pitch *p* were based on the selective light reflection phenomenon [60]. The measurements of the temperature dependence of the helical pitch *p* were done using light at normal incidence, i.e., penetrating the sample along the helical pitch. To confirm the SmC* phase existence and to identifying the observation of the half pitch or full pitch selective reflection band, the spectrum were measured at oblique incidence [61]. The *p*(T) characteristics were calculated from the equation *p* = λ_S_/*n*_av_ for SmC_A_* and *p* = λ_S_/2*n*_av_ for SmC* (the value *n*_av_ = 1.5 was estimated according to [56,62]) using data acquired for samples observed at normal incidence. The transmission spectra were acquired using a Shimadzu UV–VIS–NIR spectrometer (wavelength range of 360–3000 nm). An AMLWU7 controller with a Peltier element was used for temperature control within the range of 2–110 °C and had an accuracy of ±0.1 K. All the measurements were done during the cooling cycle.

### Dielectric spectroscopy

The frequency dispersion of the electric permittivity was measured during cooling using a Schlumberger 1260 impedance analyser in the frequency range of 10 Hz–1 MHz, keeping the temperature of the sample stable during frequency sweeps within ±0.1 K.
